# In vitro fragmentation performance of a novel, pulsed Thulium solid-state laser compared to a Thulium fibre laser and standard Ho:YAG laser

**DOI:** 10.1007/s10103-021-03495-8

**Published:** 2021-12-14

**Authors:** Lea Kraft, Ralf Petzold, Rodrigo Suarez-Ibarrola, Arkadiusz Miernik

**Affiliations:** Department of Urology, Faculty of Medicine, University of Freiburg—Medical Centre, Hugstetter Str. 55, 79106 Freiburg, Germany

**Keywords:** Holmium laser, Laser lithotripsy, Pulsed Thulium laser, Thulium fibre laser, Urinary stone, Urolithiasis

## Abstract

The aim of this work was to compare the fragmentation efficiency of a novel, pulsed Thulium solid-state laser (p-Tm:YAG) to that of a chopped Thulium fibre laser (TFL) and a pulsed Holmium solid-state laser (Ho:YAG). During the fragmentation process, we used a silicone mould to fixate the hemispherical stone models under water in a jar filled with room-temperature water. Each laser device registered the total energy applied to the stone model to determine fragmentation efficiency. Our study examined laser settings with single pulse energies ranging from 0.6 to 6 J and pulse frequencies ranging from 5 to 15 Hz. Similar laser settings were applied to explicitly compare the fragmentation efficiency of all three devices. We experimented with additional laser settings to see which of the three devices would perform best. The fragmentation performance of the three laser devices differed statistically significantly (*p* < 0.05). The average total energy required to fragment the stone model was 345.96 J for Ho:YAG, 372.43 J for p-Tm:YAG and 483.90 J for TFL. To fragment the stone models, both Ho:YAG and p-Tm:YAG needed similar total energy (*p* = 0.97). TFL’s fragmentation efficiency is significantly lower than that of Ho:YAG and p-Tm:YAG. Furthermore, we found the novel p-Tm:YAG’s fragmentation efficiency to closely resemble that of Ho:YAG. The fragmentation efficiency is thought to be influenced by the pulse duration. TFL’s shortest possible pulse duration was considerably longer than that of Ho:YAG and p-Tm:YAG, resulting in Ho:YAG and p-Tm:YAG exhibiting better fragmenting efficiency.

## Introduction

Urinary stone disease is one of the most common diseases worldwide, affecting between 3 and 12% of the population. Its prevalence appears to be increasing in industrialised countries. In the USA, the incidence of stone disease has more than doubled since the late 1970s. The situation is similar in Japan, Germany and other European countries. A nationwide survey from 1979 to 2001 in Germany shows a threefold increase in its incidence and prevalence (0.54–1.47% and 4.0–4.7%) [1]. Urolithiasis is thus considered a common condition. The USA’s prevalence is about 12%. The prevalence of wealth-related urinary stone disease is also rising in economically emerging countries. [2]

Over the last three decades, it has become the gold standard in laser lithotripsy to employ pulsed holmium solid-state laser (Ho:YAG) to treat renal, ureteral and bladder stones [3]. The most commonly used methods for intracorporeal stone disintegration are dusting and fragmenting. In general, the dusting technique refers to laser settings with low energy (0.2–0.5 J) and high frequency (15–80 Hz) that produce minute fragments that pass spontaneously [4–6]. The fragmentation technique, on the other hand, necessitates high-energy (> 1 J) and low-frequency settings (< 10 Hz) that create fragments requiring active retrieval [4, 7]. There is a substantial body of research and clinical trials demonstrating the benefits of fragmentation over dusting, and vice versa. [4, 7–9]. This emphasises the importance of providing a laser capable of both dusting and stone fragmentation [10, 11].

There has been a surge of interest in using the dusting technique for laser lithotripsy for several years. The availability of Ho:YAG with its higher frequency rates and the recently available Thulium fibre laser (TFL) with lower pulse energy settings (up to 25 mJ) and higher frequencies (up to 1600 Hz) have all contributed to this trend [12–16]. The novel, pulsed Tm:YAG solid-state laser (p-Tm:YAG) has the potential to turn the long-running controversy between competing lithotripsy techniques on its head. The performance of p-Tm:YAG in dusting has been investigated [17]. When fibres were moving at a rate of 1500 mm/min, p-Tm:YAG enabled a 12 to 17% improvement in dusting efficiency over Ho:YAG.

We compared the fragmentation performance of the p-Tm:YAG to the current gold standard Ho:YAG and the recently available TFL in this report. The fragmenting efficiency of the laser devices was compared using the same independent variables of pulse energy and pulse frequency for direct comparison. Additional laser settings, such as achieving the highest possible energy level, were chosen to assess the capability of each laser system in terms of fragmentation efficiency.

The laser emitted wavelength of each laser device, which affects the water absorption coefficient and, as a result, the optical penetration depth in water, is one parameter that may influence fragmentation efficiency. It will be left out of the debate because it is not investigated in this paper.

The peak power corresponding to the pulse duration is another feature that distinguishes these three types of laser devices used in laser lithotripsy. According to the manufacturer’s specifications, TFL’s pulse duration is up to seven times longer than that of p-Tm:YAG and 13 times longer than that of Ho:YAG, resulting in TFL’s lower peak power. This disparity in pulse duration may have major effects on fragmentation efficiency. [16, 18]. According to current knowledge, higher laser peak powers may lead to faster stone fragmentation.

## Materials and methods

The p-Tm:YAG and Ho:YAG used in the following experiments were the same as those used by Petzold et al. [17]. The TFL was used as the third laser device. Table 1 shows the specifications of all three laser devices.

**Table 1:** Comparison of the laser devices, utilised in the following experiments, in terms of general and technical specification

Parameter	Holmium solid-state laser	Thulium solid-state laser	Thulium fibre laser
Operating mode	Pulsed	Pulsed	Pulse generation by chopping CW laser beam
Abbreviation	Ho:YAG	p-Tm:YAG	TFL
Model	MEDILAS® H SOLVO® 35 – old	Evaluation model	YLR-2000-U
Manufacturer	Dornier MedTech Laser GmbH, Wessling, Germany	Dornier MedTech Laser GmbH, Wessling, Germany	IPG Photonics © IRE-Polus, Fryazino, Russia
Wavelength	2080 nm	2013 nm	1940 nm
Water absorption coefficient at 1013 bar and 37 °C	3 ${\text{mm}}^{-1}$ [14]	6.8 ${\text{mm}}^{-1}$ [19]	14 ${\text{mm}}^{-1}$ [14]
Pulse energies	0.1–3.5 J	0.1–3 J	0.025–6 J
Pulse durations	0.14–0.45 ms	0.15–1.2 ms	0.05–12 ms
Pulse frequencies	3–25 Hz	5–200 Hz	6–1600 Hz
Maximum average power	35 W	120 W	40 W

For these experiments, we used a single-use laser fibre (Dornier SingleFlex® 400 µm of Dornier MedTech GmbH) with a diameter of 400 µm.

Stone model spheres made of gypsum and glass (KMP Kugelmanufaktur Pekruhl, Germering, Germany) were used to simulate the inhomogeneous structure of kidney stones. The spheres had a diameter of 12 mm and weighed approximately 85 g each. The spheres were sawed into hemispheres of similar size. The hemispheres were submerged in room temperature water for at least 4 h before the experiments began.

**Fig. 1:**
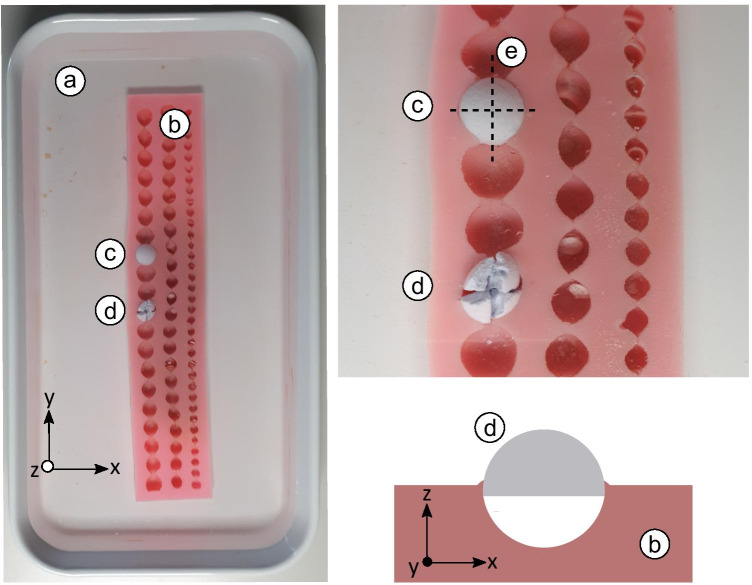
(**a**) Jar filled with room-temperature water; (**b**) silicone moulds to attach the hemispherical stone model; (**c**) hemispherical stone model attached to silicone mould; (**d**) originally attached hemispherical stone model fragmented into four pieces; (**e**) hand-guided movement of the laser fibre indicated by the dotted lines

A jar filled with room temperature water and a silicone mould to attach the hemispherical stone models formed our experimental setup (Fig. 1). The stone model was fragmented into four parts by moving the laser fibre in a cruciform path by hand. The hand-guided movement of the laser fibre was purposefully chosen in order to keep it in contact with the stone model at all times and to replicate the clinical approach to fragmenting a urinary stone. The laser fibre was adjusted in the depth of the groove created through cruciformly ablating the hemispherical stone models. An electronically operated feed, such as with an XY plotter, on the other hand, cannot ensure contact between the laser fibre tip and the stone model, which might reduce total efficacy.

During the fragmentation process, each laser system recorded the total energy required before the hemispheric stone model was fragmented into four pieces of roughly equal size. The fragmentation process was repeated five times with the same laser device at each laser setting. The mean total energy was calculated for each laser setting, yielding the mean total energies ${\text{E}}_{1}$ (Ho:YAG), ${\text{E}}_{2}$ (p-Tm:YAG) and ${\text{E}}_{3}$ (TFL).

For the fragmentation technique, we chose standard laser settings (${\text{E}}_{\text{SP}}$ = 0.6–3 J, *f* = 5–15 Hz) and the shortest possible pulse duration. All three laser devices alternated in performing five experimental runs per laser setting. The laser settings we selected are shown in Table 2.

**Table 2:** The table illustrates laser settings used by all three laser devices specified by the single pulse energy (${\text{E}}_{\text{SP}}$), the pulse frequency (f) and resulting pulse power (${\text{P}}=$ ${\text{E}}_{\text{SP}} \bullet \mathrm{ f}$). Each laser device’s pulse duration τ is indicated

${\text{E}}_{\text{SP}}$[J]	f [Hz]	P [W]	${\uptau }_{1}$(Ho:YAG) [ms]	${\uptau }_{2}$(p-Tm:YAG) [ms]	${\uptau }_{3}$(TFL) [ms]
0.6	10	6	0.22	0.174	1.2
1	10	10	0.27	0.276	1.8
2	5	10	0.37	0.648	3.9
2	10	20	0.36	0.534	3.9
3	5	15	0.45	0.81	6
3	10	30	0.44	0.792	6

Additionally, a few laser settings were chosen to measure each laser device’s capability individually (Table 3). The pulse durations varied widely between the laser devices.

**Table 3:** The table below shows laser settings, specified by the single pulse energy (${\text{E}}_{\text{SP}}$), pulse frequency (f), resulting pulse power (${\text{P}}_{\text{SP}}=$ ${\text{E}}_{\text{SP}} \bullet \mathrm{ f}$) and pulse duration (τ) of different laser devices

Laser device	${\text{E}}_{\text{SP}}$[J]	f [Hz]	P [W]	τ [ms]
Ho:YAG	1	5	5	0.27
	3.5	10	35	0.45
p-Tm:YAG	1	5	5	0.334
	3	15	45	0.81
TFL	1	6	6	1.8
	3.5	10	35	7
	6	5	30	12
	6	6.7	40.2	12

The laser fibre was cleaved before each repetition, and the energy of a single pulse ${\text{E}}_{\text{SP}}$ was measured using a laser energy meter (StarBright®, Ophir Spiricon Europe GmbH, Darmstadt, Germany). The distance between the laser fibre’s tip and the radiation-sensitive region of the pyroelectric energy meter (Ophir® FPE80BF-DIF-C) was 55 mm. A variance of less than 50 mJ was deemed permissible. Otherwise, the fibre was re-cut or a new one was used.

The statistical assessment involved calculating the mean value and standard deviation, as well as presenting the data graphically. Python (Python Software Foundation License) was used to analyse our results. The Shapiro–Wilk test was used to determine data distribution. Levene’s test was used to establish homogeneity of variances. Kruskal–Wallis *H*‑test was used to determine the impact of the laser device on total energy (*E*). Dunn’s test was used as post hoc analysis to seek any indications of a substantial difference in fragmentation efficiency between Ho:YAG, p-Tm:YAG and TFL. Each laser device’s impact on total energy (*E*) was determined using either one-way analysis of variance (ANOVA) or Kruskal–Wallis *H*-test, depending on Levene’s test and Shapiro–Wilk test. Tukey’s HSD test or Dunn’s test (based on the Bonferroni correction principle) was used for post hoc analysis. All analyses had a significance level of 0.05.

## Results

**Fig. 2:**
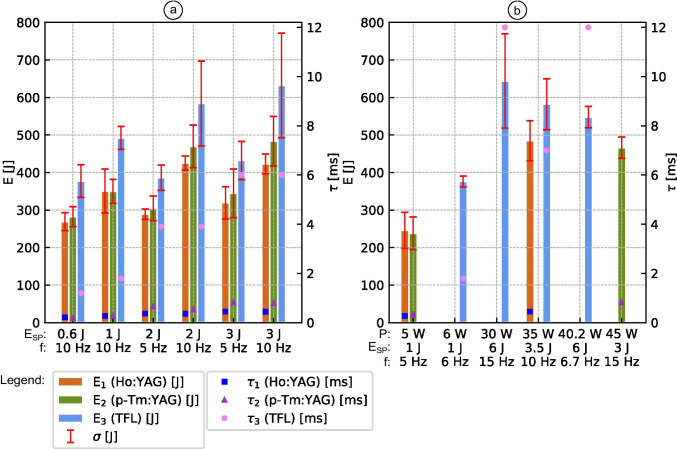
The left y-axis shows the mean total energy (${\text{E}}_{1}$, ${\text{E}}_{2}$, ${\text{E}}_{3}$) of each laser device, based on the laser settings from Table 2 (**a**) as well as Table 3 (**b**). The standard deviation (σ) of five experimental runs of each laser device for each laser settings is given as red line. The pulse durations (${\uptau }_{1}$, ${\uptau }_{2}$, ${\uptau }_{3}$) of the different laser settings in milliseconds, depending on the laser device, is placed at the right y-axis

The pulse durations of TFL are clearly longer for each laser setting compared to Ho:YAG and/or p-Tm:YAG, as the diagrams show (Fig. 2).

Figure 2a shows each laser device’s average total energy (*E*) required to fragment a hemispherical stone phantom into four pieces, based on the laser settings from Table 2 and pulse duration (*τ*). The mean total energy of Ho:YAG, p-Tm:YAG and TFL was 346 J (standard deviation: SD = 70 J), 372 J (SD = 92 J) and 484 J (SD = 125 J), respectively. Total energies recorded by p-Tm:YAG were normally distributed (*W* = 0.93, *p* = 0.05), in comparison to that of Ho:YAG (*W* = 0.92, *p* = 0.04) and TFL (*W* = 0.89, *p* = 0.004). Equal variances were assumed (*F* = 1.40, *p* = 0.25). According to Kruskal–Wallis *H*‑test, there is a significant difference between the laser devices’ main tendencies (*H* = 22.98, *p* = 1e − 05). TFL varies substantially from both Ho:YAG (*p* = 1e − 05) and p-Tm:YAG (*p* = 0.001), according to post hoc Dunn’s test. There was no substantial difference between p-Tm:YAG and Ho:YAG (*p* = 0.97).

In conclusion, at all laser settings, Ho:YAG and p-Tm:YAG need less mean total energy than TFL. To fragment the artificial stones, both Ho:YAG and p-Tm:YAG require a similar mean total energy (*p* = 0.97).

Another of our findings was that increased pulse energy and/or pulse frequency does not always imply better stone fragmentation.

In addition, we investigated whether there was any statistically significant difference between each laser device’s total energy and laser setting. Table 4 shows each laser setting’s mean total energy of Ho:YAG (${\text{E}}_{1}$), p-Tm:YAG (${\text{E}}_{2}$) and TFL (${\text{E}}_{3}$), as well as the standard deviation (SD). The total energies of each laser device’s setting were distributed normally. Equal variances were assumed for Ho:YAG (*F* = 1.27, *p* = 0.31), p-Tm:YAG (*F* = 0.52, *p* = 0.76) and TFL (*F* = 2.39, *p* = 0.07). According to one-way ANOVA, the total energy and laser settings of Ho:YAG (*F* = 15.02, *p* = 1e − 06), p-Tm:YAG (*F* = 11.78, *p* = 8e-06) and TFL (*F* = 6.89, *p* = 4e − 04) were statistically different.

**Table 4:** The mean total energy (E) and standard deviation (SD) in Joule for each laser device (Ho:YAG, p-Tm:YAG and TFL) as well as for each laser setting, specified by the single pulse energy (${\text{E}}_{\text{SP}}$) in Joule, the pulse frequency (f) in Hertz and the single pulse power (P) in Watt

${\text{E}}_{\text{SP}}$[J]	f [Hz]	P [W]	Ho:YAG		p-Tm:YAG		TFL	
			${\text{E}}_{1}$[J]	SD [J]	${\text{E}}_{2}$	SD [J]	${\text{E}}_{3}$	SD [J]
0.6	10	6	268.8	24.0	282.6	26.8	377.4	43.8
1	10	10	350.6	58.4	349.6	31.7	492.0	30.6
2	5	10	289.0	13.5	304.8	32.9	386.0	33.8
2	10	20	425.2	18.7	469.6	56.7	584.0	113.2
3	5	15	319.2	43.4	344.4	64.5	432.0	50.8
3	10	30	423.0	26.0	483.6	66.1	632.0	139.2

Post hoc Tukey’s HSD test revealed differences in each laser device’s various laser settings: For Ho:YAG, we observed significant differences between the mean total energies of laser settings 0.6 J/10 Hz and 1 J/10 Hz (*p* = 0.027), 0.6 J/10 Hz and 2 J/10 Hz (*p* = 0.001), 0.6 J/10 Hz and 3 J/10 Hz (*p* = 0.001), 2 J/5 Hz and 2 J/10 Hz (*p* = 0.001), 2 J/5 Hz and 3 J/10 Hz (*p* = 0.001), 2 J/10 Hz and 3 J/5 Hz (*p* = 0.003), 3 J/5 Hz and 3 J/10 Hz (*p* = 0.003). For p-Tm:YAG, there were significant differences between the mean total energies of laser settings 0.6 J/10 Hz and 2 J/10 Hz (*p* = 0.001), 0.6 J/10 Hz and 3 J/10 Hz (*p* = 0.001), 1 J/10 Hz and 2 J/10 Hz (*p* = 0.03), 1 J/10 Hz and 3 J/10 Hz (*p* = 0.009), 2 J/5 Hz and 2 J/10 Hz (*p* = 0.001), 2 J/5 Hz and 3 J/10 Hz (*p* = 0.001), 2 J/10 Hz and 3 J/5 Hz (*p* = 0.02), 3 J/5 Hz and 3 J/10 Hz (*p* = 0.006). For TFL, there were significant differences between the mean total energies of laser settings 0.6 J/10 Hz and 2 J/10 Hz (*p* = 0.01), 0.6 J/10 Hz and 3 J/10 Hz (*p* = 0.002), 2 J/5 Hz and 2 J/10 Hz (*p* = 0.02), 2 J/5 Hz and 3 J/10 Hz (*p* = 0.003), 3 J/5 Hz and 3 J/10 Hz (*p* = 0.02).

Figure 2b depicts each laser device’s average total energy (*E*) required to fragment the hemispherical stone model, based on the laser settings in Table 3 and pulse duration (*τ*). These laser settings were chosen to test fragmentation efficiency at each laser device’s technical limits.

Higher single-pulse energies do not inherently result in higher fragmentation efficiency when comparing the laser settings of 6 J/15 Hz/30 W, 6 J/6.7 Hz/40.2 W and 3 J/15 Hz/45 W, as shown in Fig. 2b. In general, TFL needs more energy to fragment the stone model than Ho:YAG and p-Tm:YAG, based on the mean total energy.

Taking into account that TFL’s pulse frequency at laser setting 1 J/6 Hz/6 W is 1 Hz higher than that of Ho:YAG and p-Tm:YAG at the 1 J/5 Hz/5 W laser setting; note that the Ho:YAG and p-Tm:YAG exhibit similar fragmentation efficiency, and both need less mean total energy than TFL as in Fig. 2b.

## Discussion

This study investigated the fragmentation performance of a p-Tm:YAG in comparison to a Ho:YAG and TFL. Stone fragmentation performance is known to be influenced by parameters such as the single pulse energy (${\text{E}}_{\text{SP}}$), pulse frequency (*f*) and pulse duration [7, 16, 18]. We compared the laser devices applying similar single pulse energies and pulse frequencies. The pulse duration for each setting could not be chosen to be identical due to fundamental differences in laser technology and pulse generation. Whenever possible, we were careful to select the shortest possible pulse duration for each laser device at each laser setting to enable the highest possible peak power. Additional laser settings were chosen to examine the capabilities of each laser device (e.g. high pulse energies in TFL).

Our first important finding was that the Ho:YAG and p-Tm:YAG needed less mean total energy to fragment the stone model in the same way as TFL. We made this observation in conjunction with various laser settings using pulse energies ranging from 0.6 to 3 J and frequencies ranging from 5 J to 10 Hz. From the clinical point of view, lowering the total laser energy applied to the urinary tract during an endoscopic stone removal procedure can lower the risk of thermal injuries. Advanced laser technologies, according to Williams et al. [20], allow for higher output power (energy per second), which increases the risk of thermal tissue damage caused by heating the irrigation fluid within the urinary tract. Hein et al. [21, 22] emphasise that there is thermal damage potential in combination with insufficient irrigation rates even at low-power (low-energy and/or low-frequency) settings.

Furthermore, our research discovered that the p-Tm:YAG’s fragmentation efficiency is statistically equal to that of Ho:YAG (*p* = 0.97) and outperforms the TFL’s (*p* = 0.001). To the best of our knowledge, this is the first study in urologic research to compare the fragmentation efficiency of three different laser technologies: Ho:YAG, TFL and the novel p-Tm:YAG, which is currently awaiting approval.

Secondly, we observed that TFL has a longer pulse duration and therefore lower peak power than Ho:YAG and p-Tm:YAG at all laser settings. These are the most likely causes of the lower fragmentation efficiency we observed.

Wezel et al. [18] reported that, in two different types of Ho:YAG, reducing the pulse duration from 700 to 350 µs results in more thorough stone disintegration,, two different stone compositions and two different fibre diameters.

According to Bader et al. [23], the fragmentation efficiency of a Ho:YAG at equal power settings between short and long pulse durations requires similar amounts of time before the stone models are sufficiently disintegrated. Dust weight and laser activation time served as measurement variables. They point out that there is no consensus as to what stone-particle size constitutes genuine ‘stone dust’. Dust weight is not a universal metric, since it depends on factors such as fragment size. Since we wanted to eliminate the influence of frequency, we consciously distanced our work from a time-dependent measurement variable such as laser activation time. As a result, we chose to focus solely on the effects of laser pulse energy.

Another potential drawback is employing just one type of laser device, with pulse durations varying at the same single pulse energy and pulse frequency. The long pulse had a pulse duration 1.5 to 2.6 times longer than the short pulse. For single pulse energies ≥ 1 J, the pulse durations of Ho:YAG and p-Tm:YAG were 1.5 to 6 times longer than that of TFL in our experiments.

Bell et al. [24] compared the fragmentation times of two separate Ho:YAG laser devices, one with low power and the other with high power. Regardless of pulse duration, they discovered that the high-power laser device completed the stone fragmentation procedure in half the time that the low-power laser device required. This indicates that factors other than pulse duration, such as the energy applied, may have affected fragmentation efficiency. Treatment time before fragmentation was established as one of the calculated variables. As a measurement variable, treatment time may thus be influenced more than the total energy measured by the laser device, for example, by the pedal activation time. Aldoukhi et al. [25] suggested that the relationship between laser activation time and lithotripsy time, including pedal non-activation periods, be taken into account.

Alghamdi et al. [26] recently compared three different Ho:YAG pulse shapes in terms of stone disintegration versus total operating time. The shape of the laser pulses varied at the beginning of the laser pulse. Their findings suggest that, in addition to pulse duration, pulse shape can influence fragmentation efficiency.

We also observed that to fragment a stone, more total energy is required at constant energy levels and higher frequencies. We believe that the laser fibre is harder to control at higher frequency settings: as they create more dust, visibility is reduced, thus raising the risk of tissue perforation [7].

Our experiments may have been affected by certain primarily unseen circumstances. Firstly, the pulse duration at similar laser settings varied in each laser device for technical reasons. Furthermore, dividing the spherical stone models into two hemispheres may have resulted in hemispheres of slightly different size. Finally, to obtain replicable outcomes, we used artificial stone models. The composition, crystalline structure and scale of natural human stone samples vary significantly [27].

Further experimental studies and observations of the various principles of action of these laser systems are needed to enable clinically more robust recommendations.

## Conclusion

According to our observations, the TFL’s fragmentation efficiency was significantly lower than that of Ho:YAG and p-Tm:YAG. Furthermore, the fragmentation efficiency of p-Tm:YAG proved to be very similar to that of Ho:YAG. We found the TFL’s shortest possible pulse duration to be considerably longer than that of Ho:YAG and p-Tm:YAG. That factor alone may have been the primary cause of the TFL’s poor fragmentation performance. Clinical trials are needed to confirm these experimental findings.

## Data Availability

The raw data is with the corresponding author and can be provided on request.

## Abbreviations

Ho:YAG: Pulsed, holmium:yttrium aluminium garnet solid-state laser
p-Tm:YAG: Pulsed thulium:yttrium aluminium garnet solid-state laser
TFL: Chopped, thulium fibre laser
M: Mean value
SD (σ): Standard deviation in Joule
${\text{E}}_{\text{SP}}$: Single pulse energy in Joule
f: Pulse repetition rate/pulse frequency in Hertz
P: Single pulse power in Watt

## Code availability

The computer software for statistical analysis is with the corresponding author and can be provided on request.
